# Supplementary material for: Analysis of astigmatism outcomes after horizontal rectus muscle surgery in patients with intermittent exotropia
Source: PLoS One. 2020 Oct 8;15(10):e0240026. doi: 10.1371/journal.pone.0240026 (PMC7544045; doi:10.1371/journal.pone.0240026)
Supplement: Attachment — Submitted filename: Response_to_Reviewers.docx [file pone.0240026.s003.docx]

**Responses to Reviewers’ Comments**

Review Comments to the Author

Please use the space provided to explain your answers to the questions above. You may also include additional comments for the author, including concerns about dual publication, research ethics, or publication ethics. (Please upload your review as an attachment if it exceeds 20,000 characters)

**Reviewer #1: The authors presented an original article entitled "Analysis of astigmatism outcomes after horizontal rectus muscle surgery in subjects with intermittent exotropia". This study examined the factors affecting corneal curvature change after lateral rectus recession and medial rectus resection surgery (R&R) in patients with intermittent exotropia (IXT). This was a retrospective cross-sectional study.**

My comments to the authors are:

1-In the abstract section line 30 the authors said "The factors found to affect corneal curvature change were sex, age, extent of surgery, and axial length (AL). I suggest that age is removed as the authors will mention later that age had no effect on the corneal curvature changes, lines 40-41.

🡪 Thank you for this correction. We removed “age” from line 26 of the Abstract.

2-In the methodology section the authors mentioned that the patients were divided into 2 groups (below 8 years old and 8 years or older, can the authors explain why in particular, did they choose this age to divide their patients?

🡪 Thanks for raising this valid point. We used the median as a measure of central tendency and divided our patients into two groups based on the median value, rather than the average. We then conducted our analysis based on this classification. We now clarified this point in the Methodology section, lines 90–92.

3-In the methodology section the authors mentioned that the axial lengths were divided into 2 groups (less than 23.15 mm and 23.15 mm or longer, also no explanation was given for choosing this axial length in particular.

🡪 Thanks for raising such a good point. We used the median as a measure of central tendency and divided our patients into two groups based on the median value, rather than the average. We then conducted our analysis based on this classification. We have clarified this point in the Methodology section, lines 90–92.

4-In TABLE 1: "Extent of surgery ( [5.0, 4.0], [6.0, 5.0] : above", this sentence is not clear at all, if this is the surgical dose this should be written in a clearer way.

🡪Thank you for your concern. We added Table 3 (shown below) and changed the line from "Extent of surgery ( [5.0, 4.0], [6.0, 5.0] : above )" to "Extent of surgery ( [5.0 mm/ 4.0 mm] + [6.0 mm/ 5.0 mm] : above) in Table 1.

**Table 3. Classification of extent of surgery by exodeviation prism diopter**

| **Exodeviation angle** | **Extent of surgery (LR recession/MR resection)** | **Number of patients** |
| --- | --- | --- |
| 20 PD | 5.0 mm/4.0 mm | 13 |
| 25 PD | 6.0 mm/5.0 mm | 36 |
| 30 PD | 7.0 mm/5.5 mm | 17 |
| 35 PD | 7.5 mm/6.0 mm | 10 |
| 40 PD | 8.0 mm/6.5 mm | 6 |
| 50 PD | 10.0 mm/7.0 mm | 1 |
| Total |  | 83 |

LR: lateral rectus; MR: medial rectus; PD: prism diopter

5- In the surgery analytical method: the authors divided the patients into 2 groups (recession : resection ; 5.0 mm : 4.0 mm + 6.0 mm : 5.0 mm versus above, they didn't mention the preoperative angle of deviation and how did they calculate the surgical dose.

🡪 Thank you for raising this concern. We have added a new Table 3 to show the six classification groups according to preoperative exodeviation (shown below).

**Table 3. Classification of extent of surgery by exodeviation prism diopter**

| **Exodeviation angle** | **Extent of surgery (LR recession/MR resection)** | **Number of patients** |
| --- | --- | --- |
| 20 PD | 5.0 mm/4.0 mm | 13 |
| 25 PD | 6.0 mm/5.0 mm | 36 |
| 30 PD | 7.0 mm/5.5 mm | 17 |
| 35 PD | 7.5 mm/6.0 mm | 10 |
| 40 PD | 8.0 mm/6.5 mm | 6 |
| 50 PD | 10.0 mm/7.0 mm | 1 |
| Total |  | 83 |

LR: lateral rectus; MR: medial rectus; PD: prism diopter

6- Authors have to describe in details the difference between the different ways of analyzing K-readings, astigmatism power and axis in the methodology section to be able to understand the difference between ray tracing and simulated mode.

🡪 Thank you for raising such a valid point. Anterior and posterior astigmatism, including keratometry, were calibrated in ray tracing mode. Anterior astigmatism was compared only in simulated mode. We have added this explanation to the Methodology section, lines 114-115.

7-In the results section: it is preferable to mention astigmatism in terms of power in diopters and axis in degrees, whenever they are mentioned in the entire manuscript.

🡪 Thank you for your helpful comment. We now refer to astigmatism in terms of power in diopters and to the axis in degrees, throughout the manuscript.

**Reviewer #2: Good work really but:**
1- regarding to amount of surgery we don't know about the higher limit of RR surgery and the average of recession and resection in every subset

🡪 Thank you for your helpful comment. We have added a new table with six classification groups, according to preoperative exodeviation (Table 3).

| **Exodeviation angle** | **Extent of surgery (LR recession/MR resection)** | **Number of patients** |
| --- | --- | --- |
| 20 PD | 5.0 mm/4.0 mm | 13 |
| 25 PD | 6.0 mm/5.0 mm | 36 |
| 30 PD | 7.0 mm/5.5 mm | 17 |
| 35 PD | 7.5 mm/6.0 mm | 10 |
| 40 PD | 8.0 mm/6.5 mm | 6 |
| 50 PD | 10.0 mm/7.0 mm | 1 |
| Total |  | 83 |

LR: lateral rectus; MR: medial rectus; PD: prism diopter

2- It was to be very useful if you tried to inspect the relation between the change in astigmatism and the axis of the original astigmatism, this may be more important than the change of the axis (for example; if the positive axis was 75 or 80 will this make the final increase of astigmatism less than that if the axis was 90?)

🡪 Thank you for your helpful comment. In accordance with your comment, after dividing our patients into two groups based on the median of the preoperational axis of astigmatism, the values for each group were further divided into simulated mode and ray tracing mode for comparison. The results are presented in the table below.

| **Variables** | | **Preop** |
| --- | --- | --- |
| Axis (°) (Ray tracing) | 90 ° under | 73.895 $\pm$ 15.311 |
|  | 90 ° over | 96.933 $\pm$ 6.743 |
| Axis (°) (Simulated) | 90 ° under | 77.610 $\pm$ 13.264 |
|  | 90 ° over | 96.500 $\pm$ 6.017 |

In addition, we investigated how the preoperative axis affected the postoperative change in astigmatism. The preoperative axis was divided by the median value, and the resultant groups were compared. In both ray tracing mode and simulated mode, the median preoperative axis value was 90 degrees, and the change in astigmatism in ray tracing mode increased 1 week after surgery in both the group with preoperative axis values below 90 degrees and the group with preoperative axis values of 90 degrees or more. It decreased significantly by the third month after surgery. Among subjects with an axis of less than 90 degrees, astigmatism returned to baseline—without achieving statistical significance in the paired t test—3 months after surgery. In the group of subjects with an axis of more than 90 degrees, the change in astigmatism remained statistically significant 3 months after surgery. The same result was obtained in simulated mode. Future research should study the preoperative axis affected by the vector force due to R&R surgery as well as the effects on the sclera and cornea, in greater detail. We raise these points in the revised Discussion section, lines 250–262

| **Variables** | | **Preop** | **Postop 1wk** | **Postop 3M** |
| --- | --- | --- | --- | --- |
| Astigmatism (D) (Ray tracing mode) | Axis 90° under | 1.482$\pm$1.209 | 2.133$\pm$0.960 | 1.626$\pm$0.930 |
|  | Axis 90° over | 1.292$\pm$0.681 | 2.089$\pm$0.883 | 1.673$\pm$0.747 |
| Astigmatism (D) (Simulated mode) | Axis 90° under | 1.485$\pm$1.173 | 2.151$\pm$0.907 | 1.607$\pm$0.946 |
|  | Axis 90° over | 1.372$\pm$0.657 | 2.052$\pm$0.824 | 1.718$\pm$0.687 |

3- since that maximum age was just 28y and most of patients were around the average age (9.5y), the paper can't conclude that age isn't an important factor, there should be a wide range of ages and a considerable number of adults.

🡪 Thank you for your helpful comment. We agree with your opinion. In accordance with your comment, the ages of the 83 patients are now depicted in a bar graph as follows:

Age

Number (N)

| Age (Years) | Number(N) |
| --- | --- |
| 3 | 1 |
| 4 | 1 |
| 5 | 8 |
| 6 | 12 |
| 7 | 10 |
| 8 | 15 |
| 9 | 8 |
| 10 | 8 |
| 11 | 1 |
| 12 | 5 |
| 13 | 2 |
| 14 | 2 |
| 15 | 2 |
| 16 | 1 |
| 18 | 2 |
| 20 | 1 |
| 23 | 1 |
| 26 | 1 |
| 28 | 2 |

In the Discussion section, we mention that age had no effect because the range was narrow; however, this was not the result in all age groups, indicating a bias. We now discuss this bias in the Discussion section. The bias can be reduced in future research by subdividing the larger age group into smaller groups. Future studies should also adopt a prospective design.

4- the myopic shift can't be determined unless one had did cyclorefraction in every stage of the assessment.

🡪 Thank you for your helpful comment. We checked all patients with cycloplegic refraction after the prism cover test because the average age was 9.5 years old. We have added this information to the methodology section, lines 109–111: “Furthermore, SE was evaluated after the prism cover test by a single examiner (LSY) using cycloplegic refraction. See Table 2.”

5- the myopic shift can't be physiological (line 252) since the period is just 3 months.

🡪 Thank you for your helpful comment. As you mention, it is difficult to explain the myopic shift change by old age. However, younger patients could be particularly affected by the physiological changes inherent to axial lengthening. We therefore changed the sentence as follows: “The myopic shift that remained 3 months after the operation might be explained by the fact that the patients who underwent muscle resection and recession showed higher scleral effects in comparison with the effects observed in nonsurgical patients, whose mean age was 9.55 years. This age coincides with the age at which myopia increases physiologically [23–25].” (lines 267–270).
